# Singaporean Dementia Caregivers’ Preference for Care Recipients’ Life-Extending Interventions

**DOI:** 10.1093/geroni/igaa057.1760

**Published:** 2020-12-16

**Authors:** Chetna Malhotra, Hazirah Mohamad, Truls Østbye, Kathryn Pollak, Bharathi Balasundaram, Sungwon Yoon, Rahul Malhotra, PISCES study Group PISCES study Group

**Affiliations:** 1 Duke-NUS Medical School, Singapore, Singapore; 2 Duke University, Durham, North Carolina, United States; 3 Duke University School of Medicine, Durham, North Carolina, United States; 4 Changi General Hospital, Singapore, Singapore

## Abstract

Family caregivers of older adults with severe dementia are decisive in use of potentially life-extending interventions for their care recipients. We conducted in-depth interviews with 26 caregivers of community-dwelling older adults with severe dementia in Singapore to assess their preferences for intravenous (IV) antibiotics for a life threatening infection, tube feeding, and cardiopulmonary resuscitation (CPR), and reasons thereof. Most caregivers’ (77%) end-of-life care goal was ‘no life extension’. Yet, 80%, 60% and 45% preferred IV antibiotics, tube feeding and CPR, respectively, as they: 1) perceived letting go by withholding interventions as unethical, 2) felt they had no choice, deferring to health care providers (HCPs), 3) wanted to alleviate suffering, and 4) desired trying minimally invasive (and potentially withdrawable) interventions. There was discordance between caregivers’ end-of-life care goal and preferences for life-extending interventions. HCPs can suggest intervention options that concur with caregivers’ end-of-life care goal, and use a shared decision-making approach. Part of a symposium sponsored by the Aging Among Asians Interest Group.

